# UV-Triggered Drug Release from Mesoporous Titanium Nanoparticles Loaded with Berberine Hydrochloride: Enhanced Antibacterial Activity

**DOI:** 10.3390/molecules29071607

**Published:** 2024-04-03

**Authors:** Fanjiao Zuo, Boyao Wang, Lizhi Wang, Jun He, Xilong Qiu

**Affiliations:** 1 Tianjin State Key Laboratory of Modern Chinese Medicine, Tianjin University of Traditional Chinese Medicine, Tianjin 301617, China; zuofanjiao@163.com; 2 School of Chinese Materia Medica, Tianjin University of Traditional Chinese Medicine, Tianjin 301617, China; wangboyao2007@163.com; 3 School of Traditional Chinese Medicine, Tianjin University of Traditional Chinese Medicine, Tianjin 301617, China; lzhwang_2009@163.com; 4 Haihe Laboratory of Modern Chinese Medicine, Tianjin 301617, China

**Keywords:** mesoporous titanium nanoparticles, UV-controlled, berberine hydrochloride, antibacterial effect

## Abstract

Mesoporous titanium nanoparticles (MTN) have always been a concern and are considered to have great potential for overcoming antibiotic-resistant bacteria. In our study, MTN modified with functionalized UV-responsive ethylene imine polymer (PEI) was synthesized. The characterization of all products was performed by different analyses, including SEM, TEM, FT-IR, TGA, XRD, XPS, and N_2_ adsorption-desorption isotherms. The typical antibacterial drug berberine hydrochloride (BH) was encapsulated in MTN-PEI. The process exhibited a high drug loading capacity (22.71 ± 1.12%) and encapsulation rate (46.56 ± 0.52%) due to its high specific surface area of 238.43 m^2^/g. Moreover, UV-controlled drug release was achieved by utilizing the photocatalytic performance of MTN. The antibacterial effect of BH@MTN-PEI was investigated, which showed that it could be controlled to release BH and achieve a corresponding antibacterial effect by UV illumination for different lengths of time, with bacterial lethality reaching 37.76% after only 8 min of irradiation. The minimum inhibitory concentration (MIC) and minimum bactericidal concentration (MBC) of the nanoparticles have also been studied. The MIC of BH@MTN-PEI was confirmed as 1 mg/mL against *Escherichia coli* (*E. coli*), at which the growth of bacteria was completely inhibited during 24 h and the concentration of 5 mg/mL for BH@MTN-PEI was regarded as MBC against *E. coli*. Although this proof-of-concept study is far from a real-life application, it provides a possible route to the discovery and application of antimicrobial drugs.

## 1. Introduction

Infections are considered to be the most serious complication associated with human health and need to be addressed urgently. Among them, bacterial infection has always been one of the most serious health issues that people have faced, and the discovery and application of antimicrobial drugs are the keys to treating this kind of infection [1,2]. However, a number of important bacterial pathogens, such as *Escherichia coli* (*E. coli*), *Pseudomonas aeruginosa* (*P. aeruginosa*), and *Staphylococcus aureus* (*S. aureus*), have developed significant resistance in clinics with the widespread use of antimicrobial drugs [3,4,5,6,7,8]. Among them, *E. coli*, which is a member of the *Enterobacteriaceae* family, can cause diarrhea or extraintestinal diseases, newborn meningitis, abdominal sepsis, septicemia, and urinary tract infections, although it lives as a harmless commensal in the colon in the majority of cases [9,10,11,12]. The growing antimicrobial resistance in *E. coli* has been reported worldwide, and the *Enterobacteriaceae* family has been ranked by the WHO as the bacteria that constitutes the greatest threat to human health [13,14]. Therefore, it is imperative to provide a sustainable strategy to prevent and control *E. coli*, as well as to minimize the adverse effects of antibacterial agents on the ecological environment and human health.

In this situation, we urgently need an innovative antibacterial strategy to fight drug-resistant pathogens, especially multi-drug-resistant strains. Recently, the development of nanotechnology has provided significant ways for designing and creating artificial nanoplatforms with controlled release capabilities to reduce the amount of antibacterial agent and reinforce the antibacterial strategy, including inorganic nanoparticles [15,16,17], liposomes [18,19,20], nanogels [21,22], polymeric micelles [23,24], etc., which showed excellent and unique properties as well as significant effects in addressing drug resistance [25]. It is nanomaterials’ small particle size, large specific surface area, and ease of surface functionalization that allow them to be used as antimicrobial delivery agents to improve interactions with pathogens and membranes, increase drug solubility, and improve drug biocompatibility.

Typically, mesoporous titanium nanoparticles (MTN) as a representative of inorganic nanomaterials, which are suitable to be applied for the purpose of a photocatalytic antibacterial agent, have a significant potential for precision nanomaterials because of their low toxicity, chemical stability, strong photocatalytic activity, and large surface area [16,26,27,28]. The antibacterial activity of MTN is attributed to the production of reaction oxygen species (ROS), and it is generally accepted that UV irradiation is critical for ROS generation and the antibacterial activity of MTN in spite of several activities that can be triggered in the dark [29]. At the same time, the generation of ROS could effectively decompose the organic compounds attached to the surface of MTN, providing theoretical support for UV-triggered drug release [30]. The MTN with rutile/anatase heterojunctions prepared by Qin et al. had good photocatalytic antibacterial effects on *E. coli*. It was explained by nanoparticles’ large specific surface area, large pore size, and special crystalline phase structure, which resulted in an increased adsorption capacity for bacteria and a large amount of ROS to kill the bacteria under the light condition [31]. Chen et al. demonstrated that MTN@SiO_2_ hybrid materials could generate ROS under UV and visible light, which migrated into bacterial cells, leading to cell damage [32].

Berberine hydrochloride (BH) is a broad-spectrum natural antibacterial agent that mainly exists in plants such as *Berberis aquifolium*, *Coptis chinensis,* and *Phellodendron amurense* [33]. The biological activities of BH have been reported, including anticancer, anti-inflammatory, antioxidant, and excellent antibacterial effects on various Gram-positive bacteria and Gram-negative bacteria [33,34,35]. However, because of its poor solubility and low bioavailability, the application of BH has been limited for a long time. Thus, researchers have devoted themselves to exploring ways to improve the stability and bioavailability of BH. Li et al. created an oil-in-water nanoemulsion system for BH that was anticipated to improve the relative oral bioavailability of BH in rats and serve as an alternate oral formulation for BH [36]. The Yang group designed chitosan-coated nano-liposome carriers loaded with BH, which showed better stability and a slow release effect [37].

**Scheme 1:**
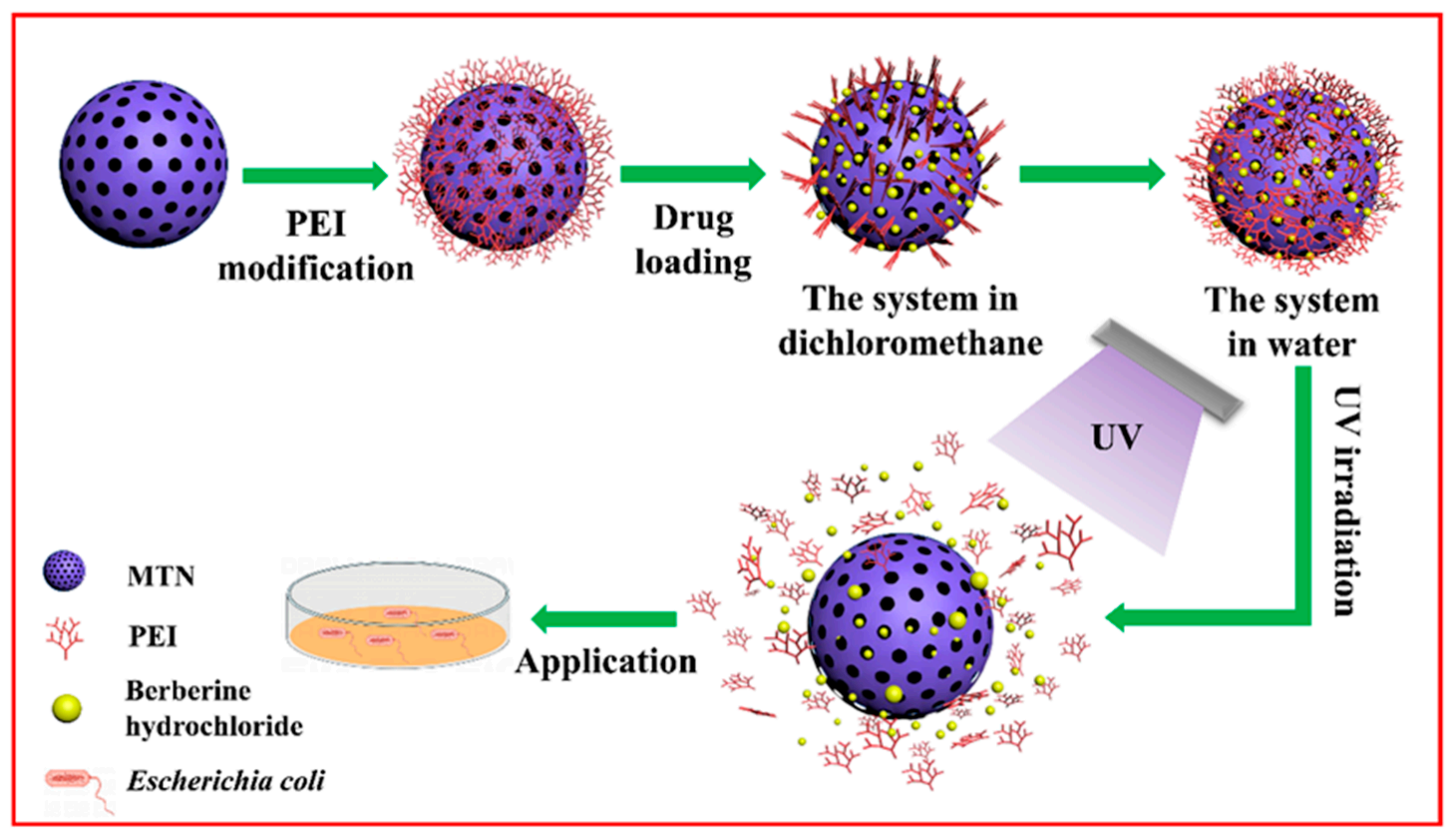
Schematic representation of the preparation process of BH-loaded MTN-PEI and its release of cargos in response to UV light.

In this work, we adopted a facile sol-gel method to synthesize MTN with a small size and well-defined mesoporous structure. Through using ethylene imine polymer (PEI) as a gatekeeper, we succeeded in coating a thin PEI layer on the surface of the MTN with the purpose of keeping drugs from leaking prematurely. The property of the PEI layer to be destroyed by UV irradiation was critical for controlling the release of BH, which we used in *E. coli* to investigate the synergistic antibacterial capability of BH and MTN-PEI, as well as the controllable antimicrobial capacity of BH@MTN-PEI under UV conditions (Scheme 1). This UV-controlled release drug delivery system will improve drug solubility, thus making drugs as effective as possible in their applications. What is novel is that the system will not only solve the problem of drug solubility and resistance but also achieve a combined antibacterial effect, providing new carrier options and ideas for antibacterial research.

## 2. Results and Discussion

### 2.1. Preparation and Characterization of MTN, MTN-PEI, and BH@MTN-PEI

**Figure 1:**
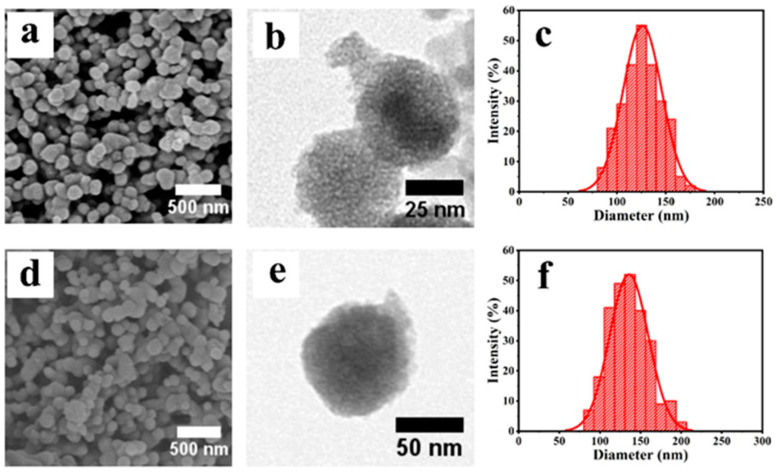
SEM images of MTN (**a**) and MTN-PEI (**d**). TEM images of MTN (**b**) and MTN-PEI (**e**). The size distribution of MTN (**c**) and MTN-PEI (**f**).

In this study, MTN was synthesized using a template-directed sol-gel method with the surfactant F127 as the template and TIP as the source of titanium, followed by the removal of the template by extracting the samples with absolute ethanol. Examination of the MTN by SEM indicated (Figure 1a) the successful fabrication of MTN with a monodisperse spherical shape with an average particle size of ca. 126 nm in diameter. TEM revealed (Figure 1b) the existence of ordered mesoporous structures on the surfaces of the MTN, which were formed by the initial formation and subsequent removal of F127 spherical micelles. A feature that was confirmed (Figure S1) from the wide-angle powder X-ray diffraction (XRD) patterns displayed by the freshly obtained MTN.

The synthesis of MTN functionalized with PEI onto its outside surface was prepared, and positively charged PEI was deposited on the surface of negatively charged MTN through electrostatic adsorption, as described in detail in the Experimental Section. The morphology of MTN-PEI analyzed by SEM (Figure 1d) showed that MTN-PEI are mainly homogeneous and spherical in shape, with an average size of ca. 137 nm in diameter, which was also confirmed by TEM (Figure 1e). This also indicated that the modification of PEI did not affect the dispersion or morphology of the nanoparticles. In this study, a typical triblock (PEO/PPO/PEO) polymer F127 was used as a structure-directing agent to form internal hydrophobic and external hydrophilic spherical micelles in an ethanol medium, and the TIP reaction rate was strictly controlled to synthesize MTN with a small particle size [38].

The microcrystalline structure of MTN was verified by small-angle powder XRD (Figure S1). The intermediate MTN showed no apparent diffraction peaks, reflecting the amorphous characteristic of MTN [38]. The framework of amorphous MTN exhibited a twisted octahedral structure, with structural units characterized by short-range ordering and long range disorder [39]. In addition, the atomic arrangement in amorphous MTN could be described as an assembly of short staggered chains of edge and vertex-connected Ti-O octahedral units [40].

**Figure 2:**
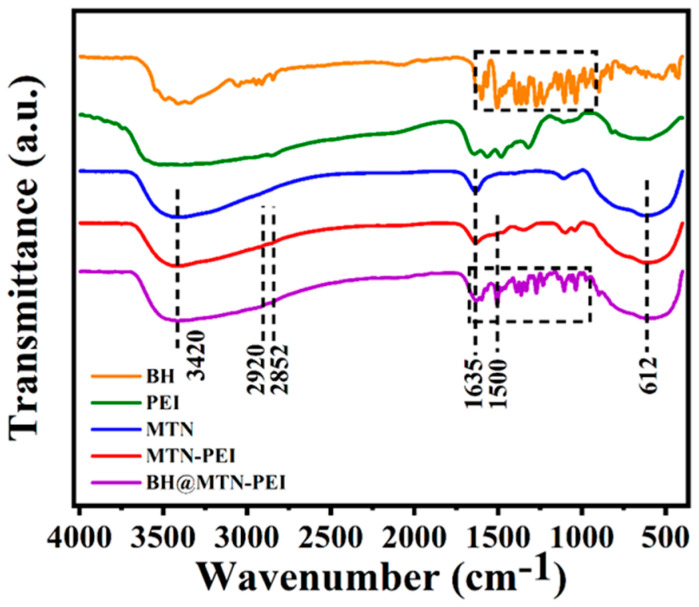
FT-IR curves of BH (origin), PEI (green), MTN (blue), MTN-PEI (red), and BH@MTN-PEI (purple).

FT-IR spectroscopy (Figure 2) has been used to monitor the progress of MTN’s functionalization based on MTN. There were peaks at 612 cm^−1^ and 1635 cm^−1^ in the spectrum of MTN, which were the characteristic absorption of a typical vibration peak of Ti-O-Ti and H_2_O, respectively [38,41]. MTN had a strong adsorption band at 3420 cm^−1^, which was the stretching vibration of O-H resulting from a large amount of absorbed water on the surface of MTN. After modification of MTN with PEI, the absorption bands at 2919 cm^−1^, 2852 cm^−1^ and 1500 cm^−1^ were shown, providing further evidence of PEI functionalization on the surfaces of MTN through electrostatic interaction [42]. Those peaks in the spectrum of BH@MTN-PEI at 1600 cm^−1^, 1598 cm^−1^, 1505 cm^−1^, 1463 cm^−1^, 1365 cm^−1^, 1275 cm^−1^, 1232 cm^−1^, and 1034 cm^−1^ belonged to the characteristic absorption of BH [43,44].

**Figure 3:**
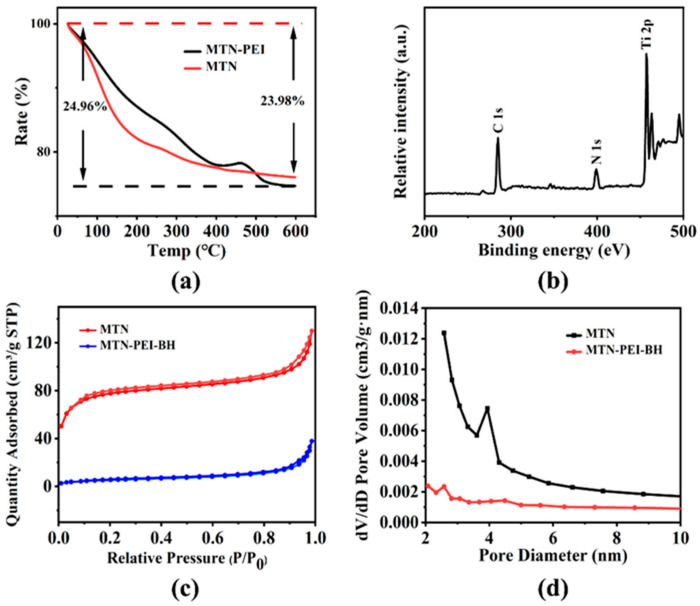
(**a**) TGA curves of MTN (red) and MTN-PEI (black). (**b**) XPS pattern of MTN-PEI (black). (**c**) The nitrogen adsorption-desorption isotherms of MTN (red) and BH@MTN-PEI (blue). (**d**) The pore size distribution curves of MTN (black) and BH@MTN-PEI (red).

Since MTN was thermally stable, the weight loss in the TGA curves (Figure 3a) at high temperatures was attributed to the loss of its surface groups. There was an approximately 23.98% weight loss in MTN, which might be ascribed to the burning out of water in the internal structure as well as on the surface [45]. Then, a number of PEIs were successfully assembled on the surface of the MTN, as inferred by the spare 0.98% weight loss difference between MTN and MTN-PEI. There were three well-resolved peaks of C 1s, N 1s and Ti 2p in the XPS analysis (Figure 3b). The peak center of the C 1s spectrum was 287 eV (Figure S3a), while the peak energy of the N 1s spectrum generated from PEI was 531 eV (Figure S3b). In the Ti 2p spectrum (Figure S3c), the binding energies of 457 and 463 eV were assigned to Ti 2p_3/2_ and Ti 2p_1/2_, respectively [46]. In addition, ζ-potential experiments indicated that PEI-modified MTN had positive surface charges and the MTN possessed negatively charged surface charges (Figure S2, Table S3). The absolute value of the ζ-potential of the bare MTN was found to be >20 mV, an observation that indicated that the synthesized nanoparticles maintained a certain stability in an aqueous solution. The ζ-potential of MTN after coating modification was ca. 7.15 mV, which could be accounted for by the PEI’s abundance of positively charged amino groups [47].

Surface areas and pore widths were confirmed by Brunauer-Emmett-Teller (BET) and Barrett-Joyner-Halenda (BJH) analyses, which clearly revealed the physical properties (Figure 3c, Table S1 in ESI). It transpired that the bare MTN exhibits characteristic Type IV BET isotherms with surface areas of 238.43 m^2^/g, which is attributed to the presence of cylindrical nanopores. The Brunauer-Emmett-Teller (BET) model worked out the specific surface (S_BET_) value of MTN and BH@MTN-PEI, as shown in Table S1. A narrow BJH pore size distribution was indicated in accordance with the steep condensation step, and the pore volumes of MTN and BH@MTN-PEI are displayed in Table S1. The nitrogen adsorption-desorption isotherms further indicated that the synthesized MTN had mesoporous structures.

### 2.2. In Vitro Release Evaluation

**Figure 4:**
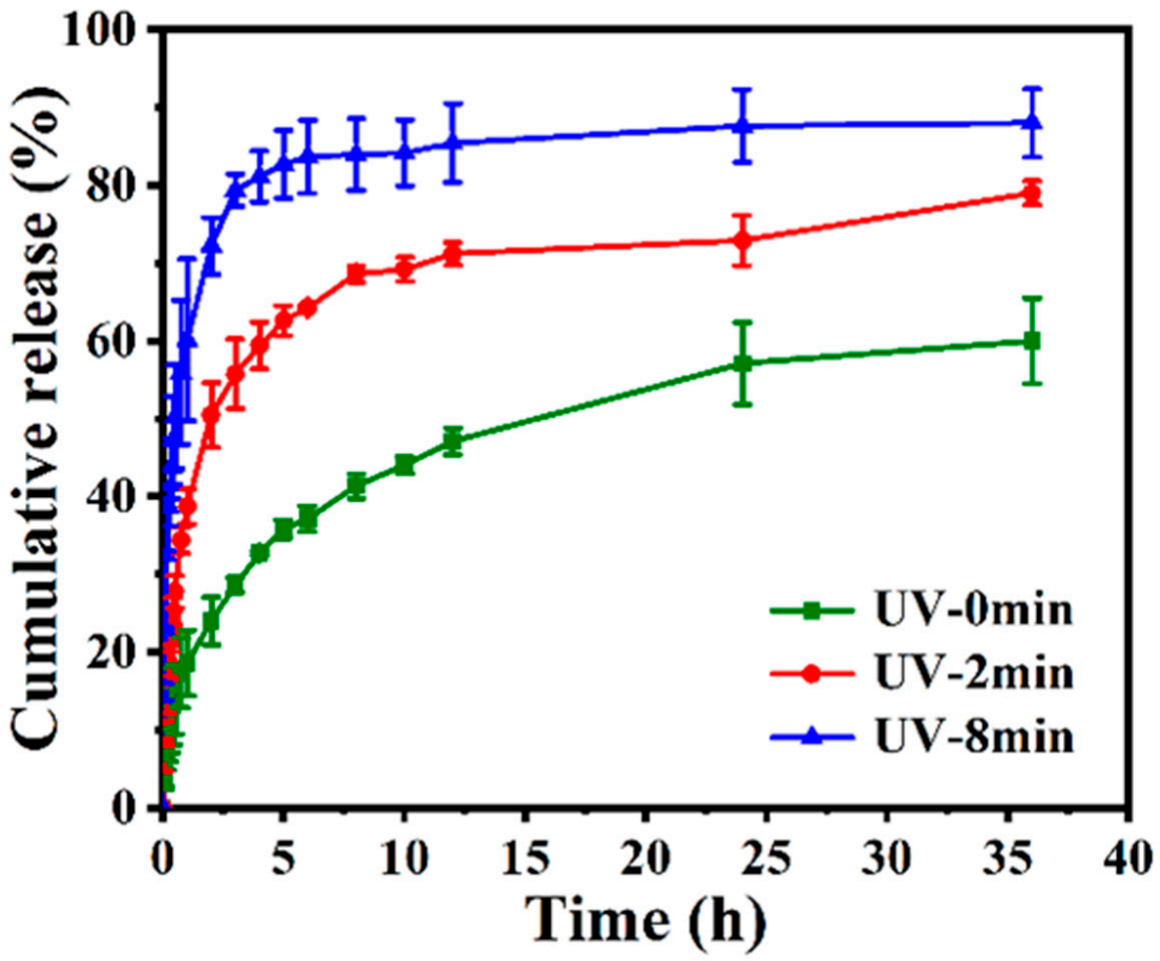
In vitro release profile of BH@MTN-PEI following different UV irradiation times (0, 2, and 8 min).

Prior to investigating the UV-controlled release of drugs, loading evaluation was determined by a UV spectrophotometer. Nitrogen adsorption-desorption isotherms (Figure 3c) and the results of BH loading efficiency (22.13 ± 1.49%) and encapsulation (Table S2) efficiency (52.39 ± 2.32%) were consistent with each other. According to Wang et al.’s explanation, the hydrophilic layer that PEI formed on the carrier’s surface was the crucial factor by which drugs were loaded and packaged [48]. Next, BH, which had poor solubility and was effective against *E. coli*, was used as a model drug. The much lower BH released from the BH@MTN-PEI nanomaterial in the absence of stimulation indicated that PEI has been successfully capped on MTN again (Figure 4). It was hampered by the hydrophilic dendritic macromolecule PEI having its branches opened in the aqueous phase. Additionally, it revealed that the cumulative release of BH was the highest under the condition of UV-irradiation for 8 min. The cumulative amount of BH under different irradiation times indicated the UV-responsive ability of the MTN-PEI system because the PEI coated on MTN nanoparticles was destroyed after UV irradiation, dramatically increasing the cumulative release of BH.

### 2.3. Antibacterial Study

**Figure 5:**
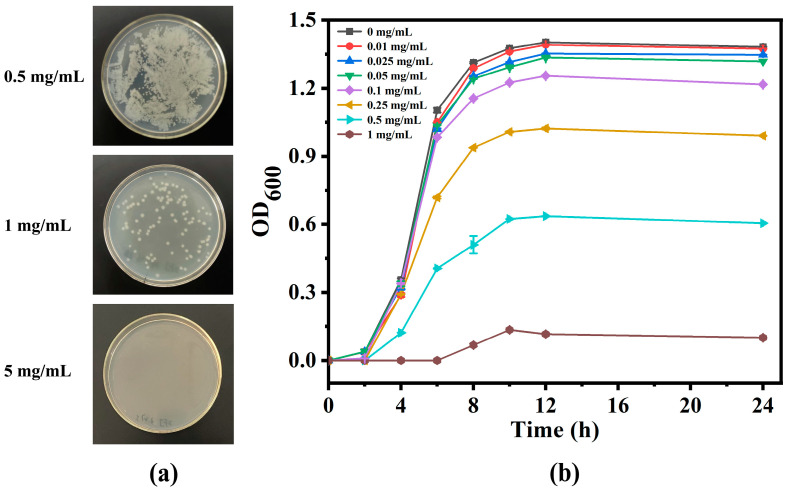
(**a**) Photographs of representative plates in the MBC test of *E. coli*. (**b**) The evolution of the OD_600_ of bacteria in the presence of BH@MTN-PEI with different concentrations.

MIC and MBC experiments were conducted on *E. coli* to evaluate the antibacterial activity of nanoparticles. The experimental group exposed to UV for 8 min was the one used for testing, as they had the best release effect in the in vitro release experiment. In order to eliminate the impact of drugs released during the co-cultivation process between nanoparticles and *E. coli* on absorbance measurement, nanoparticles were pretreated with drug release. The antibacterial curves (Figure 5) recorded the evolution of OD_600_ during the growth process of *E. coli* in Luria–Bertani broth (LB) with different concentrations of nanoparticles, which showed that the antibacterial activity of BH@MTN-PEI increased with their concentration. The MIC of BH@MTN-PEI was confirmed as 1 mg/mL against *E. coli*, at which point the growth of bacteria was completely inhibited for 24 h. The antibacterial activities were further evaluated via colony-counting methods. The bacteria cultured for 24 h in LB containing various concentrations of BH@MTN-PEI were taken out and 10-fold gradient diluted to cast the agar plates, and then cultured for another 24 h. The representative plates of 10-fold diluted *E. coli* cultured at various concentrations are shown in Figure 5. The CFUs decrease sharply as the concentrations of antibacterial agents increase. No colony was observed when the antibacterial agent concentration reached 5 mg/mL for BH@MTN-PEI, which was regarded as the MBC against *E. coli*.

**Figure 6:**
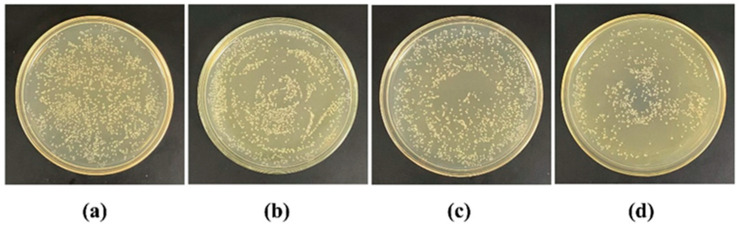
Photographs illustrating the antibacterial activity of the BH@MTN-PEI under different UV irradiations: (**a**) control, (**b**) 0 min, (**c**) 2 min, and (**d**) 8 min.

Using the spread plate method, we investigated the antibacterial effects of MTN-PEI and BH@MTN-PEI against *E. coli* under UV irradiation (Figure S4). Both MTN-PEI and BH@MTN-PEI had antibacterial effects, with the latter having a stronger effect than the former under the same irradiation period, indicating the combination of BH and MTN-PEI has a synergistic antibacterial effect [49,50]. We further evaluated the controllability of the drug delivery system by releasing the drug after different illumination periods and applying it to *E. coli* (Figure 6). The results indicated a UV-controllable amount of BH was released, corresponding to the illumination duration. In these experiments, the samples that were lit for 8 min exhibited the strongest antibacterial effects (the bacterial lethality was 37.76%) because more PEI were destroyed, resulting in the release of more usable drug molecules.

## 3. Materials and Methods

### 3.1. Materials

Titanium isopropoxide (TIP) (97%), pluronic block co-polymer F127, PEI (99%), BH, and dichloromethane were purchased from Shanghai Yien Chemical Technology Co., Ltd. (Shanghai, China). Anhydrous methanol and anhydrous ethanol were purchased from Tianjin Jiangtian Chemical Technology Co., Ltd. (Tianjin, China). The phosphate-buffered saline (PBS) solution and dialysis bag were purchased from Beijing Solarbio Science & Technology Co., Ltd. (Beijing, China). Sodium chloride was purchased from Tianjin Fengchuan Chemical Reagent Technology Co., Ltd. (Tianjin, China). Yeast extract power was purchased from Sinophar Chemical Reagent Co., Ltd. (Beijing, China). Ttyptone was purchased from Shanghai yuanye Bio-Technology Co., Ltd. (Shanghai, China). Ager was purchased from BioFroxx. Double distilled water (ddH_2_O) was purified from a Millipore purification system. *E. coli* was purchased from Tiangen Biotech (Beijing) Co., Ltd. (Beijing, China).

### 3.2. Methods

#### 3.2.1. Preparation of MTN

The MTN was prepared through a sol-gel method according to a modified literature procedure [38,48]. Firstly, 8.0 g of F127 was added to 200 mL of absolute ethanol at 40 °C while the stirring speed was 600 rpm. Then, 1.2 mL of ddH_2_O was added to the solution when the F127 was completely dissolved in ethanol. After that, 3.7 mL of TIP was slowly added to the solution drop by drop via injection at ambient temperature with vigorous stirring, and the solution was further stirred for another 45 min. During this hydrolysis process, the clear solution turned into a milky white suspension. After stewing for 15 h at ambient temperature, the precipitation was collected by centrifugation. The precipitation was washed with absolute ethanol several times until the supernatant was clear, and the products obtained were dried at 60 °C for 12 h. Finally, the samples were dispersed in 200 mL of absolute ethanol and stirred for 2 h at 70 °C. After the centrifugation, the precipitation was re-dispersed in 200 mL of absolute ethanol and extracted by the same method. After repeating this process three times, the samples were dried at 60 °C for 12 h. In order to remove the structure-directing agent thoroughly, we washed the products again in the same way.

#### 3.2.2. Preparation of MTN-PEI

The fabrication of functionalized MTN was simply achieved by a mixture of MTN and PEI [48]. Typically, 0.5 g PEI and 0.1 g MTN were added to 100 mL of PBS (pH = 7.4) and dispersed evenly by sonication for 10 min. Next, the mixed solution was continuously stirred for 10 h at room temperature. The samples were collected from the solution by centrifuging at a speed of 5000 rpm and dried for 12 h at 60 °C. The resulting samples were named MTN-PEI.

### 3.3. Characterization

Scanning electron microscopy (SEM) images were collected on a Hitachi SU8020 electron microscope operating at 20 kV. Transmission electron microscopy (TEM) images were collected on a JEM-2100 EX microscope (Japan) operated at 100 kV. Zeta potential was measured by a Zetasizer Nano ZS90 (Britain). Fourier transform infrared (FT-IR) spectra were collected on a Bruker Fourier spectrophotometer using KBr pellets. Thermogravimetric analysis (TGA) was taken with a NETZSCH STA 449F5 operated at 10 K/min in the air. The nitrogen adsorption–desorption isotherm was measured by TriStar II 3020 3.02. Specific surface areas were calculated from the adsorption data in the low-pressure range using the Brunauer-Emmett-Teller (BET) model. Pore sizes were determined following the Barrett-Joyner-Halenda (BJH) method. An X-ray diffraction (XRD) pattern was collected on a BRUCKER D8 ADVANCE. X-ray photoelectron spectroscopy (XPS) was measured using a Thermo escalab 250Xl.

### 3.4. Preparation of BH-Loaded MTN-PEI

Typically, 20.0 mg MTN was added to 10 mL dichloromethane solution containing 10.0 mg BH and then dispersed completely through ultrasonic mixing. After stirring continuously at room temperature for 24 h, the nanosuspension was centrifugated (8000 rpm, 10 min) to remove free BH. The above supernatant and washing dichloromethane solution were collected and used for measuring the content of BH by UV-vis spectrophotometer at 345 nm [51]. The samples were collected and dried at 60 °C, denoting them as BH@MTN-PEI. The BH loading efficiency (LE) and encapsulation efficiency (EE) were calculated according to the following equation, where W is the weight:

$$\mathrm{LE}\%=\frac{\left[W(\mathrm{initial}\;\mathrm{BH})-W(\mathrm{BH}\;\mathrm{in}\;\mathrm{supernatant})\right]}{\left[W(\mathrm{BH}@\mathrm{MTN}-\mathrm{PEI})\right]}\times 100\%$$

$$\mathrm{EE}\%=\frac{\left[W(\mathrm{initial}\;\mathrm{BH})-W(\mathrm{BH}\;\mathrm{in}\;\mathrm{supernatant})\right]\;}{\left[W(\mathrm{initial}\;\mathrm{BH})\right]}\times 100\%$$

### 3.5. UV-Controlled Drug Release Experiments

The in vitro drug release experiment was performed as follows: 10.0 mg BH@MTN-PEI samples and 1 mL PBS (pH = 7.4) were placed into a dialysis membrane bag (cutting off molecular weight was 8000–14,000). Soon afterwards, the dialysis bag was immersed in 100 mL of PBS (pH = 7.4) in a shaker under gentle stirring at 37 °C. A 60 W lamp was used as a UV light source, which was located 20 cm above the release solution and irradiated vertically downwards [52,53]. The different durations of UV irradiation were set to 0 min, 2 min, and 8 min. A certain amount of solution was extracted at designated time intervals, and an equal amount of fresh PBS was immediately added to the original solution so as to maintain the volume of 100 mL. The amount of BH released in the media was measured by UV-vis spectrophotometry, which appeared at 345 nm.

### 3.6. Bioactivity Studies of Antibacterial Nanoplatforms In Vitro

The antibacterial activity of the BH@MTN-PEI was studied using *E. coli*, which is responsible for many infections in daily life, served as a model target microorganism for antibacterial tests. Before the test, all glassware was sterilized via autoclaving at 120 °C for 20 min. The strain was taken out to gradually adapt to the room temperature environment and inoculated onto fresh solid culture medium using the streaking method, placing them upside down in a constant temperature incubator for cultivation. After that, single colonies were inoculated into 4 mL of LB) and incubated overnight at 37 °C in a constant temperature shaker to obtain logarithmic growth of *E. coli* for subsequent experiments.

The antibacterial properties were assessed quantitatively using the minimum inhibitory concentration (MIC) and minimum bactericidal concentration (MBC) methods. The MIC was based on the turbidity of the bacterial suspension, which was determined by measuring the optical density at 600 nm using a spectrophotometer, and the MBC was determined through colony counting [15]. *E. coli* was cultured in LB and diluted to a concentration of 2 × 10^6^ colony-forming units per milliliter (CFU/mL) approximately at 37 °C with constant agitation at 220 rpm. Different concentrations of nanoparticle solutions were added to the *E. coli* suspension, and the OD_600_ of aliquots obtained at a preset interval was recorded. The concentration that completely inhibited bacterial growth within 24 h was determined as the MIC. The bactericidal activities were studied for test tubes containing nanoparticle solutions above MIC. Aliquots taken from the tubes cultured for 24 h were 10-fold serially diluted, and 100 μL of each was plated on agar plates. Following another 24 h of incubation at 37 °C, the number of bacterial colonies on the plates was counted. MBC was determined as the minimum concentration at which 99% of bacteria were killed within 24 h.

The BH@MTN-PEI nanoparticles were weighed at 2.0 mg and dispersed in 1 mL of PBS solution at 37 °C, and then the sample solution was exposed to UV illumination for different times (0, 2, and 8 min), respectively. Adjusting the *E. coli* suspension to 8 × 10^4^ CFU/mL, 1 mL of *E. coli* suspension was taken out and mixed with the processed sample solution. The mixture was incubated at 37 °C for 5 h with shaking at 220 rpm so that the bacteria and the drug released from nanoparticles could fully contact each other. After that, 100 μL of the treated solution was spread on the nutrient medium and incubated at a constant temperature (37 °C) for 24 h. The colonies on the plate were counted and photographed for records. Bacteria without any samples were used as controls. The bacterial lethality (BL) was calculated according to the following equation, where N is the colony number:

(1) $$\mathrm{BL}\%=\left[1-\frac{N(\mathrm{experimental}\;\mathrm{group})}{N(\mathrm{control}\;\mathrm{group})}\right]\times 100\%$$

## 4. Conclusions

In the present work, a novel proof-of-concept MTN antibacterial nanoplatform based on functionally modified MTN nanoparticles and PEI-based nanogates was successfully designed and loaded by BH for the highly efficient inhibition of *E. coli*. MTN, as the main component of the nanocarrier, not only loaded the antibacterial reagent with excellent drug loading capacity but has also been recognized as a traditional semiconductor photocatalyst for its antimicrobial candidate. The results of in vitro drug release experiments have confirmed that MTN-PEI has good controlled-release ability, laying a foundation for subsequent antibacterial applications. The synergistic antibacterial capability of BH conjugated with MTN-PEI and the possible viability of MTN-PEI in light-controlled drug release were investigated by the spread plate method. Our results made it known that the drug delivery system (BH@MTN-PEI) displayed a great antibacterial effect, which could improve BH’s solubility and bioavailability. The MTN carrier with UV-controlled valves combined the advantages of well-drug loading and UV-controlled release, expecting to provide scientific guidance for promoting a much broader application of antimicrobial materials.

## Data Availability

Data are contained within the article and Supplementary Materials.

## Supplementary Materials

The following supporting information can be downloaded at: https://www.mdpi.com/article/10.3390/molecules29071607/s1, Scheme S1: Schematic representation of drug loading mechanism; Scheme S2: Schematic diagram of PEI damage with increasing UV irradiation time; Figure S1: XRD pattern of MTN; Figure S2: Zeta potential of MTN and MTN-PEI; Figure S3: XPS patterns of C 1s (a), N 1s (b), Ti 2p (c); Figure S4: Photographs illustrating the antibacterial activity of the MTN-PEI and BH@MTN-PEI under 5 min UV irradiation; Table S1: Nitrogen adsorption/desorption isotherms characterization parameters of MTN and BH@MTN-PEI, respectively; Table S2: Loading efficiency and encapsulation efficiency of BH@MTN-PEI; Table S3: Zeta potential of MTN and MTN-PEI, refs. [54,55].
