# Supplementary material for: UV-Triggered Drug Release from Mesoporous Titanium Nanoparticles Loaded with Berberine Hydrochloride: Enhanced Antibacterial Activity
Source: Molecules. 2024 Apr 3;29(7):1607. doi: 10.3390/molecules29071607 (PMC11013668; doi:10.3390/molecules29071607)
Supplement: Supplementary file 1 [file molecules-29-01607-s001.zip › molecules-2936832-supplementary.pdf]

UV-Triggered Drug Release from Mesoporous Titanium Nanoparticles Loaded with Berberine Hydrochloride: Enhanced Antibacterial Activity

Fanjiao Zuo [1], Boyao Wang [2], Lizhi Wang [3], Jun He [1,4,*] and Xilong Qiu [3,*]

1 Tianjin State Key Laboratory of Modern Chinese Medicine, Tianjin University of Traditional Chinese Medicine, Tianjin 301617, China; zuofanjiao@163.com
2 School of Chinese Materia Medica, Tianjin University of Traditional Chinese Medicine, Tianjin 301617, China; wangboyao2007@163.com
3 School of Traditional Chinese Medicine, Tianjin University of Traditional Chinese Medicine, Tianjin 301617, China; lzhwang_2009@163.com
4 Haihe Laboratory of Modern Chinese Medicine, Tianjin 301617, China
* Correspondence: hejun673@163.com (J.H.); qxlong@tjutcm.edu.cn (X.Q.)

1. Antibacterial effect of the MTN-PEI and BH@MTN-PEI

Before the test, all glassware was sterilized via autoclaving at 120°C for 20 min. The MTN-PEI and BH@MTN-PEI nanoparticles were weighed 2.0 mg and dispersed in 1 mL PBS solution at 37°C, and then the sample solution was mixed with 1 mL of *E. coli* suspension (3.6×10^5 CFU/mL). Exposing to UV illumination for 5 min, the mixture of was incubated at 37°C for 5 h with shaking. After that, 100 μL of the treated solution was spread on the nutrient medium and incubated at constant temperature (37°C) for 24 h. Bacteria without any samples was used as control.

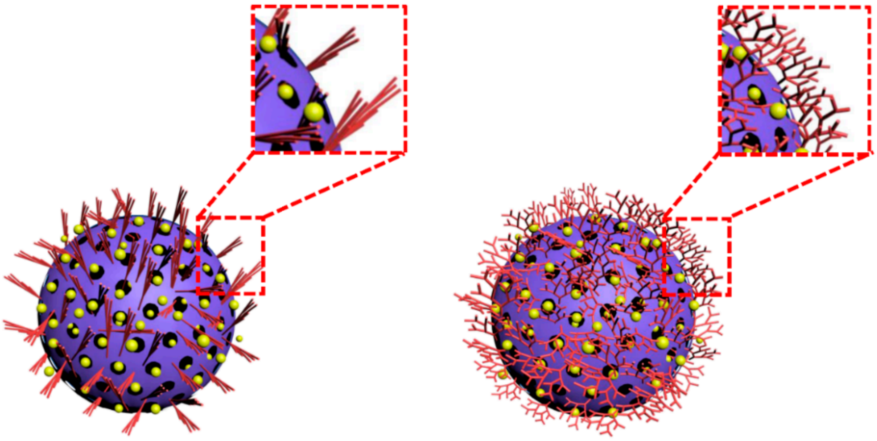

Scheme S1. Schematic representation of drug loading mechanism.

The drug was successfully loaded due to the hydrophilic characteristic of PEI, a typical hydrophilic macromolecular polymer in which dendritic branches are constricted in less polar dichloromethane, exposing the pores of the MTN and making the drug molecules exceptionally easy to load. However, in aqueous solution, the dendritic branches are open, allowing for controlled drug release while preventing premature drug leakage.

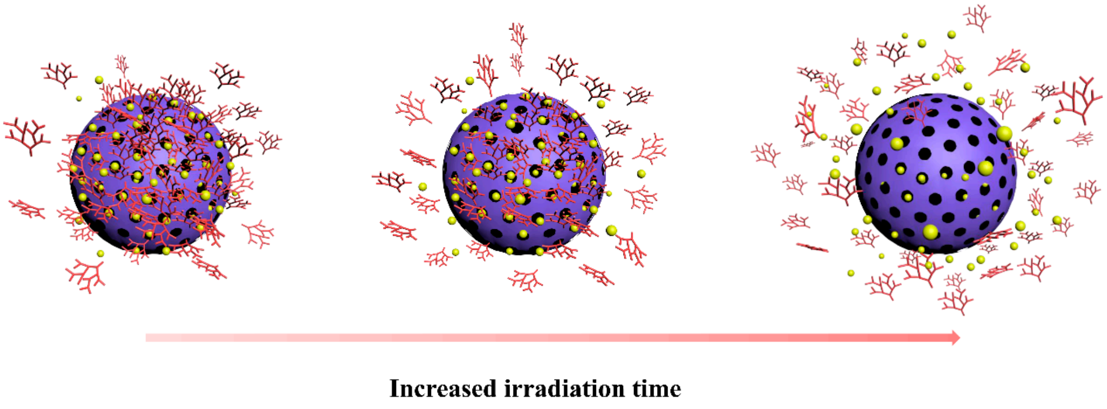

Scheme S2. Schematic diagram of PEI damage with increasing UV irradiation time

UV can selectively destroy the amino groups in PEI, resulting in the breakdown of the PEI layer on the MTN surface, and the degree of PEI layer destruction is time dependent[1]. As a result, the loaded drug release hindrance decreases and cumulative drug release increases.

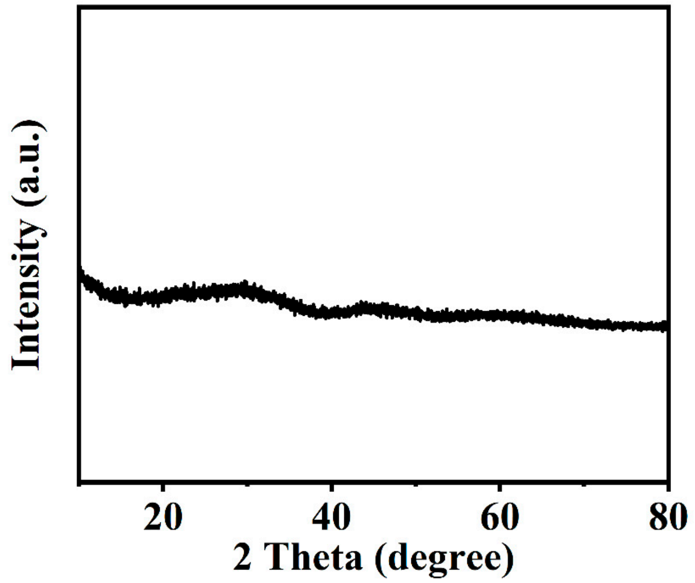

Figure S1. XRD pattern of MTN.

By using wide-angle powder XRD, the microcrystalline structure of MTN was confirmed. The intermediate MTN displayed no discernible diffraction peaks, illustrating the amorphous nature of MTN[2].

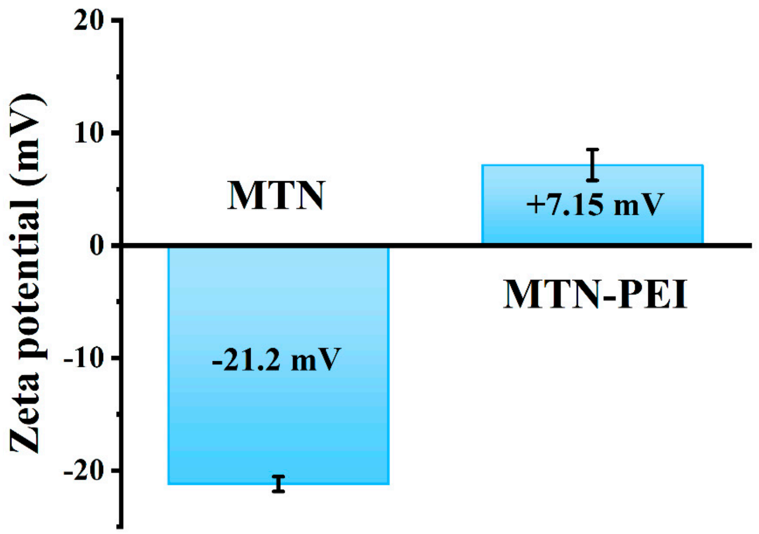

Figure S2. Zeta potential of MTN and MTN-PEI.

The suspension of MTN was found to be a typical titanium dioxide sol with a negative zeta potential value of -21.20 ± 0.54 mV, which might be attributed to the strong polarity

of the Ti-O bond in MTN, which dissociated water adsorbed on the surface to create hydroxyl groups[3].

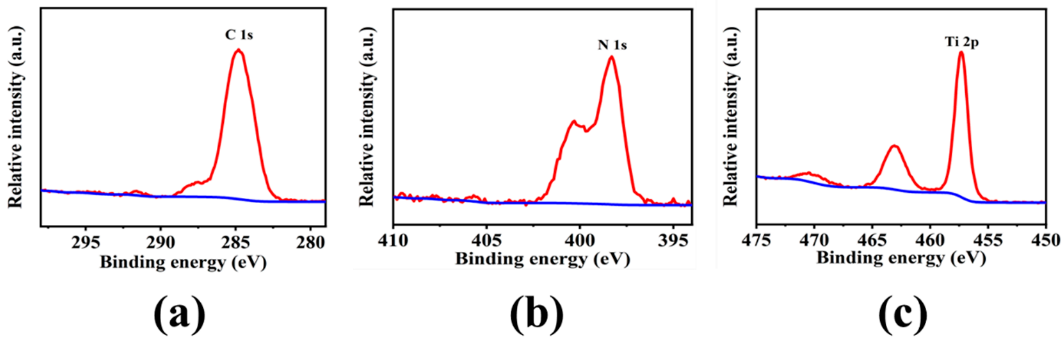

Figure S3. XPS patterns of C 1s (a), N 1s (b), Ti 2p (c).

Ti 2p XPS showed peaks at 457 and 463 eV due to peaks for Ti $2p_{3/2}$ and Ti $2p_{1/2}$, respectively. The C 1s spectrum showed peak centered at 287 eV, while the N 1s spectrum showed peaks at 531 eV.

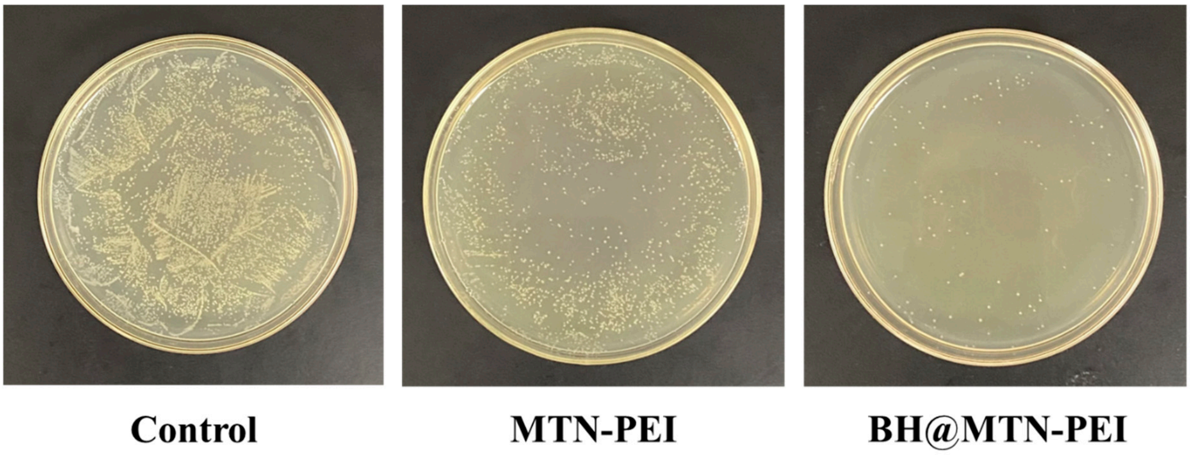

Figure S4. Photographs illustrating the antibacterial activity of the MTN-PEI and BH@MTN-PEI under 5 min UV irradiation.

The number of colonies in the plates showed that both MTN-PEI and BH@MTN-PEI had antimicrobial effects, and the comparison of the two showed the synergistic antimicrobial effects of BH and MTN-PEI.

Table S1. Nitrogen adsorption/desorption isotherms characterization parameters of MTN and BH@MTN-PEI, respectively.

	**BET surface area (m^2/g)**	**Total pore volume (cm^3/g)**	**Average pore diameter (nm)**
**MTN**	238.43	0.13	4.31
**BH@MTN-PEI**	18.73	0.05	2.58

There was a considerable decrease in specific surface area and total pore volume following drug loading, indicating that BH was successfully absorbed into the carriers' pores.

Table S2. Loading efficiency and encapsulation efficiency of BH@MTN-PEI.

	**loading efficiency**	**encapsulation efficiency**
**BH@MTN-PEI**	21.58%	47.14%
	23.82%	46.40%
	22.73%	46.14%
	22.71 ± 1.12%	46.56 ± 0.52%

The MTN-PEI's BH loading efficiency and encapsulation efficiency, respectively, were found to be 22.13 ± 1.49% and 52.39 ± 2.32%, which were determined by UV spectrophotometer. The higher BH loading efficiency of the nanoparticles were assigned to its higher specific surface area and pore volume.

Table S3. Zeta potential of MTN and MTN-PEI.

	MTN	MTN-PEI
**Zeta potential (mV)**	-20.50	+6.52
	-21.30	+8.74
	-21.80	+6.20
	-21.20 ± 1.12	+7.15 ± 1.38

References

1. Hong, H.; Lee, S.; Kim, T.; Chung, M.; Choi, C., Surface modification of the polyethyleneimine layer on silicone oxide film via UV radiation. *Appl Surf Sci* **2009,** 255, (12), 6103-6106.
2. Jia, T.; Zhang, J.; Wu, J.; Wang, D. L.; Liu, Q. Z.; Qi, Y. F.; Hu, B.; He, P.; Pan, W. G.; Qi, X. M., Synthesis amorphous TiO_2 with oxygen vacancy as carriers transport channels for enhancing photocatalytic activity. *Mater Lett* **2020,** 265.
3. Martín, A.; Morales, V.; Ortiz-Bustos, J.; Pérez-Garnes, M.; Bautista, L. F.; García-Muñoz, R. A.; Sanz, R., Modelling the adsorption and controlled release of drugs from the pure and amino surface-functionalized mesoporous silica hosts. *Microporous and Mesoporous Materials* **2018,** 262, 23-34.
